# Case-finding and genetic testing for familial hypercholesterolaemia in primary care

**DOI:** 10.1136/heartjnl-2021-319742

**Published:** 2021-09-14

**Authors:** Nadeem Qureshi, Ralph Kwame Akyea, Brittany Dutton, Steve E Humphries, Hasidah Abdul Hamid, Laura Condon, Stephen F Weng, Joe Kai, for the FAMCAT study, Paul Roderick

**Affiliations:** 1 Centre for Academic Primary Care, School of Medicine, University of Nottingham, Nottingham, UK; 2 Centre for Cardiovascular Genetics, Institute of Cardiovascular Science, University College London, London, UK; 3 Department of Primary Care Medicine, Faculty of Medicine, Universiti Teknologi MARA, Sungai Buloh, Malaysia; 4 Associate Director, Cardiovascular and Metabolism, Janssen Research & Development, High Wycombe, UK

**Keywords:** hyperlipidemias, genetics, electronic health records

## Abstract

**Objective:**

Familial hypercholesterolaemia (FH) is a common inherited disorder that remains mostly undetected in the general population. Through FH case-finding and direct access to genetic testing in primary care, this intervention study described the genetic and lipid profile of patients found at increased risk of FH and the outcomes in those with positive genetic test results.

**Methods:**

In 14 Central England general practices, a novel case-finding tool (Familial Hypercholetserolaemia Case Ascertainment Tool, FAMCAT1) was applied to the electronic health records of 86 219 patients with cholesterol readings (44.5% of total practices’ population), identifying 3375 at increased risk of FH. Of these, a cohort of 336 consenting to completing Family History Questionnaire and detailed review of their clinical data, were offered FH genetic testing in primary care.

**Results:**

Genetic testing was completed by 283 patients, newly identifying 16 with genetically confirmed FH and 10 with variants of unknown significance. All 26 (9%) were recommended for referral and 19 attended specialist assessment. In a further 153 (54%) patients, the test suggested polygenic hypercholesterolaemia who were managed in primary care. Total cholesterol and low-density lipoprotein-cholesterol levels were higher in those patients with FH-causing variants than those with other genetic test results (p=0.010 and p=0.002).

**Conclusion:**

Electronic case-finding and genetic testing in primary care could improve identification of FH; and the better targeting of patients for specialist assessment. A significant proportion of patients identified at risk of FH are likely to have polygenic hypercholesterolaemia. There needs to be a clearer management plan for these individuals in primary care.

**Trial registration number:**

NCT03934320.

## Introduction

Familial hypercholesterolaemia (FH) is one of the most common inherited disorders. The heterozygote form of FH is estimated to have a prevalence of 1 in 250.^1^ Left untreated, these individuals are at increased risk of premature coronary heart disease (CHD).^2–4^ Treatment with lipid-lowering treatment, like statins, can dramatically reduce this risk.^3 5^ However, the majority of patients remain undiagnosed and untreated.^2 6 7^

International guidelines recommend the identification of FH using various case-finding tools.^2 8 9^ In the UK, the 2008 National Institute for Health and Care Excellence (NICE) FH guidelines recommended identifying possible FH cases in primary care using the Simon-Broome (S-B) criteria.^6^ In addition to the established case-finding approaches, we have developed a novel case-finding tool (Familial Hypercholetserolaemia Case Ascertainment Tool, FAMCAT).^10^

Once an individual is identified at possible risk of FH, guidelines recommend the individual should be genetic tested to confirm the diagnosis.^9 11^ Currently, this testing is not offered in primary care but in specialist care.^12^ The most recently developed genetic test for FH uses next-generation sequencing (NGS) diagnostic assay. In addition to identifying FH-causing variants, this more comprehensive analysis identifies individuals with genetic variants of unknown significance (VUS) and those with minor variants that collectively can suggest the individual has a polygenic cause for hypercholesterolaemia.^13^

We offered direct access to FH genetic testing in primary care, as part of a study to optimise the FAMCAT case-finding tool. We sought to identify the genetic and lipid profile of patients found at increased risk of FH, and the outcomes in those identified with FH-causing genetic variants or VUS in primary care.

## Methods

### Study population and procedure

In this multicentre, non-randomised, intervention study, 14 general practices, in the catchment areas of two UK lipid specialist services, expressed an interest to participate from May 2017 to November 2019, with a total practice population of 193 589. A range of urban, rural and suburban areas were invited to take part in the study based on their English index of multiple deprivation score, an official measure of material deprivation, to ensure a diverse practices were recruited.^14^

Participating general practices were given a 1 hour face-to-face introductory session on identifying FH based on NICE FH guideline, and demonstration of how to use the FAMCAT FH case-finding tool to perform electronic search of patient records and identify eligible patients for the study. The FH case-finding tool was then installed on all general practice computers. Fifty study participation packs, for patients, were despatched to each practice.

Nominated practice administrators first performed electronic health record search to identify patients, aged 18 years and above, with a cholesterol reading (n=86 219 or 44.5% of total practices’ population). The FAMCAT1 case-finding tool was applied to these patients’ records by the practice administrator (based on the lower threshold of FAMCAT1 algorithm: probability of FH of 1 in 500).^10^ This identified 3375 at possible risk of FH of whom the first 336 consenting to completion of Family History Questionnaire (FHQ), researcher access to their clinical records and genetic testing in their practice, were recruited.

The research assistant notified the practice administrator which of the recruited patients to invite for genetic tests at phlebotomy clinics in the practice. In line with NICE FH guideline, a repeat cholesterol test was performed at the same time. As part of the invitation, participants were given an information leaflet describing the nature of the test. The genetic test was conducted by the Bristol NHS FH Genetic Laboratory (CPA Ref: 2907) using a NGS diagnostic assay. A simplified summary of the genetic test results was sent to both the participant and their nominated general practitioner (GP).

Participants were eligible if they were registered with a participating general practice, able to give written informed consent, aged 18 years or over, had a serum cholesterol recorded in their electronic health records and without a previous diagnosis of FH.

### Clinical data

Clinical data on the recruited participants were collected at baseline through automated electronic healthcare record extraction, manual review of electronic health records by a research assistant and self-reported FHQ of CHD.^15^

### Outcomes

#### Genetic outcome measures

*Genetically confirmed FH* was defined as carrying a definitive pathogenic variant in *LDLR* (18 exons), *APOB* (28 exons), *PCSK9* (12 exons) and homozygosity in *LDLRAP1* (9 exons) as reported on the laboratory test report.

VUS are variants that cannot be definitively classified as pathogenic or benign. All identified variants were independently assessed by three senior experts in FH genetic testing (MW—clinician scientist, SEH—cardiovascular geneticist, AW—lipidologist) using internationally agreed criteria published by the American College of Medical Genetics guidelines.^16^ These assessors determined if variant classifications are ‘definitely not’ and ‘likely not pathogenic’ (class 1 and 2), ‘VUS’ (class 3) and ‘likely’ and ‘definitely pathogenic’ (class 4 and 5). The assessors had not been involved in the design of the project or the analysis of these data.

*Polygenic hypercholesterolaemia risk score* (PHR score) is based on the combined weighted effect of 12 common low-density lipoprotein (LDL)-cholesterol raising single nucleotide polymorphisms (SNPs) in 11 genes.^13^ Full details of the SNPs are provided in online supplemental table 1. Using a weighted score, each individual was assigned to the appropriate decile.^13^ With a PHR score in decile 1–3 designated as a low likelihood of polygenic hypercholesterolaemia, a 6th–10th decile score designated as a high likelihood of polygenic hypercholesterolaemia and 4th and 5th decile score as intermediate likelihood.^17^

10.1136/heartjnl-2021-319742.supp1 Supplementary data

Those patients with FH confirmed on genetic test or with unclear results (VUS) were recommended for specialist referral after further discussion with their GP. If the test did not identify FH-causing variant but only indicated a high polygenic hypercholesterolaemia score, GP was informed of patient’s predisposition to raised cholesterol and recommended that they are recalled for cardiovascular risk assessment and patient advised that the practice will be in touch to arrange a cardiovascular health check. Finally, remaining patients were informed of the negative genetic test results and sent a healthy lifestyle leaflet.

### Biochemical outcome measures

All available information on lipid profiles of participants (total cholesterol, LDL-cholesterol, HDL-cholesterol and triglycerides) were extracted from patients’ records. The latter included lipoprotein(a) (Lp(a)) levels when available in the local laboratory.

### Process measures

This included the participants’ recruitment rate as defined by participant completing FHQ. Following genetic testing, we also calculated the referral rate and noted participants attending specialist referral. Also, from reviewing hospital medical records of patients seen in specialist care, we identified outcome of clinical assessment and those patients offered cascade testing to relatives.

### Patient and public involvement

Our patient and public co-applicant was involved in the design and conduct of this study. The patient representatives participated in interpreting the study results and assisted with the plain English summary.

### Statistical analysis

Primary outcome and process measures were presented descriptively. Continuous normally distributed variables were described by the mean and SD while continuous non-normally distributed variables were described by the median and IQR. Categorical variables presented as count and percentage. Wilcoxon rank-sum test for continuous data and Fisher’s exact test for categorical data were used to compare cholesterol profiles and statin prescribing between groups.

In order to complete 300 genetic tests, we planned to recruit 345 patients, assuming 13% of patient will not complete genetic testing.

## Results

The 14 recruited general practices’ index of deprivation ranged from 1 (most deprived) to 10 (least deprived) with the median deprivation score of 7 (IQR 6.25) and 5 general practices with scores below 5. Further details on the recruited practices are presented in online supplemental table 2.

**Figure 1:**
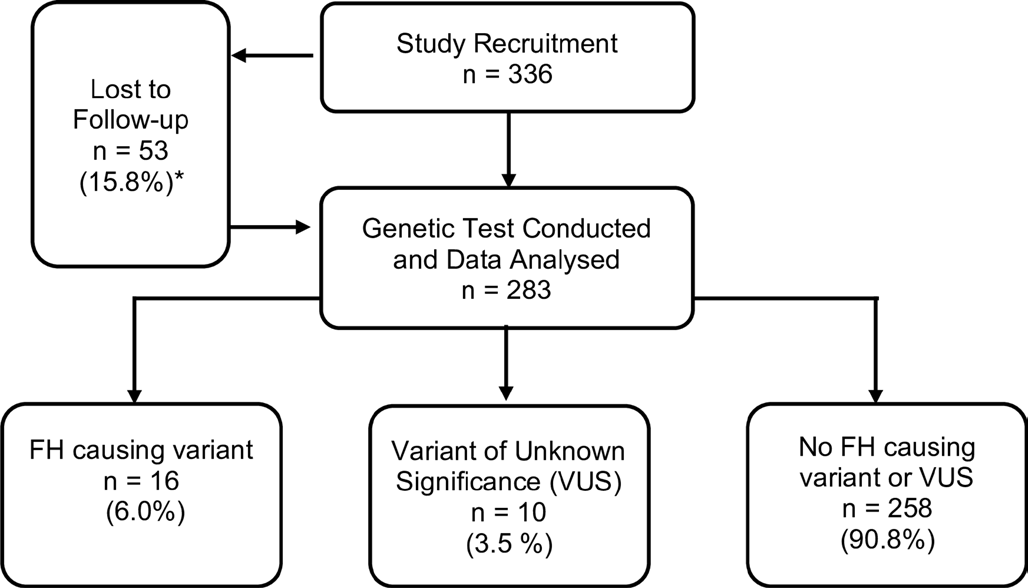
Study flow diagram. FH, familial hypercholesterolaemia. *Lost to follow up includes: 42 participants did not respond to genetic test invite and 11 left the practice before genetic test invite was sent.

**Figure 2:**
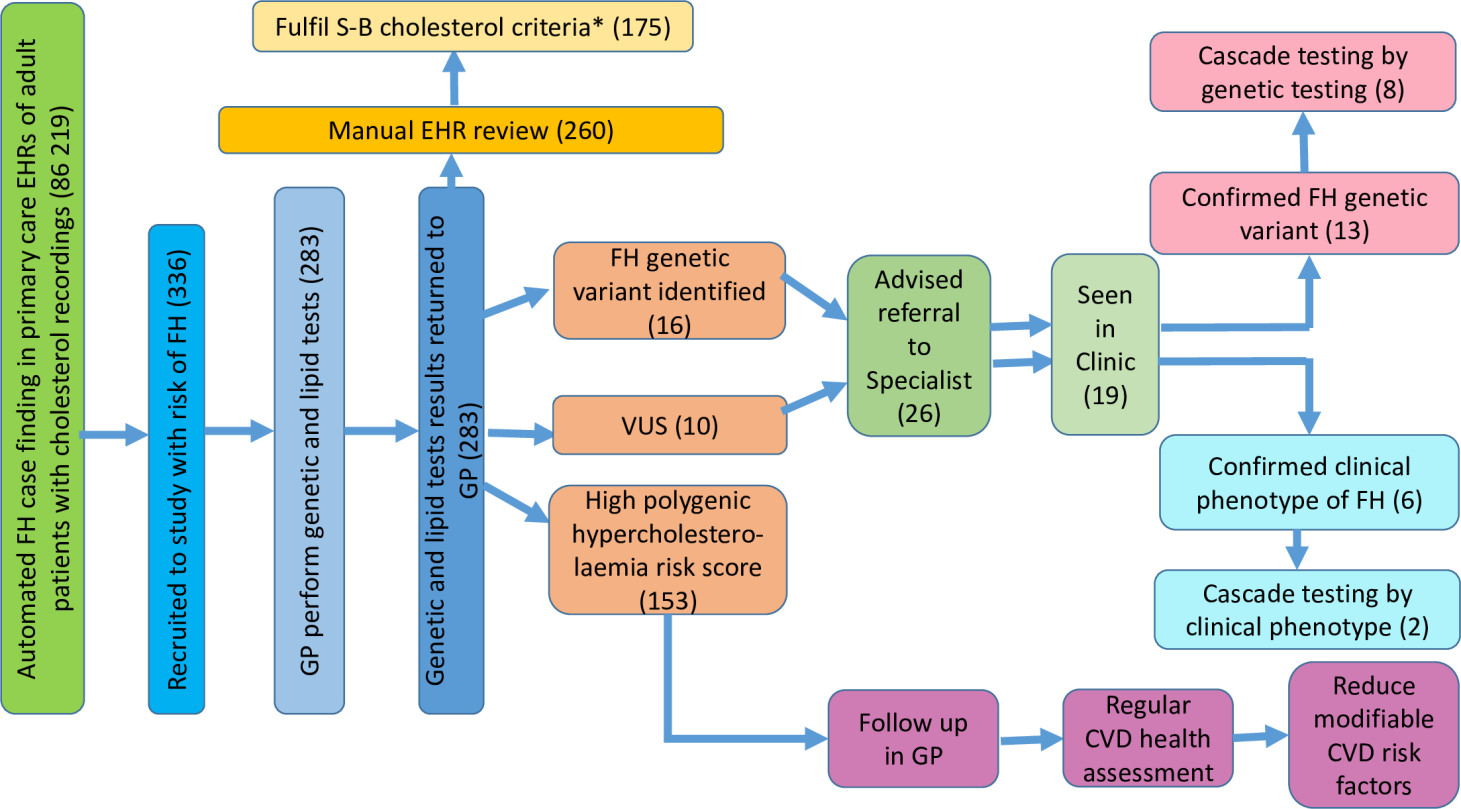
Graphical display of key findings. *Ever had total cholesterol >7.5 or LDL-C >4.9 mmol/L. CVD, cardiovascular disease; EHR, electronic health record; FH, familial hypercholesterolaemia; GP, general practitioner; S-B, Simon-Broome; VUS, variants of unknown significance.

As indicated in figures 1 and 2, of 336 consenting patients at risk of FH, 283 (84.2%) attended for genetic testing in primary care and 26 (9%) of the 283 participants tested were recommended referral for specialist assessment (16 with a confirmed FH-causing variant and 10 with VUS). During the study period, one of these patients, with genetically confirmed FH, died from cardiovascular disease. Full details of the variants that were identified are listed in online supplemental tables 3 and 4. All identified variants were reviewed by three experts. They agreed that all of the FH-causing variants were correct, and that none of VUS variants could be reclassified as pathogenic.

Of the 26 advised referral for FH-causing variant or with VUS, by completion of the study, 19 were seen by specialists (figure 2). Of the 13 with FH-causing variants (genetically confirmed FH) seen by specialist, cascade screening to relatives was commenced in 8 families. The other six patients, with VUS results were managed based on their clinical phenotype and two of these were offered cascade screening. Additional details on process outcomes are given in online supplemental table 5. A further 153 participants (54.1% of those tested) had a high polygenic hypercholesterolaemia score (deciles 6–10). The decile distribution of the whole sample is presented in online supplemental table 6 and online supplemental figure 1.

Electronic health records were available for 260 of the 283 participants who had had genetic testing. These 260 participants had a similar age-sex profile to the 76 that were lost to follow-up (online supplemental table 7). The lipid profile demonstrated raised cholesterol levels with 175 (67%) patients fulfilling S-B cholesterol criteria (table 1). Further in 93 of the participants, Lp(a) levels were available with a median of 156.8 (IQR 85–492) and 28 (10.8%) with Lp(a) above reference range (>300 mmol/L). Although no arcus cornealis was identified in general practice records, 3 of the 19 patients, seen in hospital, were found by the specialist to have arcus cornealis. No patient was found to have tendon xanthoma.

**Table 1:** Profile of study participants where manual electronic health records available (n=260)

Variable	Participants with no previous FH diagnosis (n=260)
Age in years, mean (SD)	56.3 (11.4)
Females, n (%)	180 (69.2)
Highest total cholesterol level, median (IQR)	7.6 (6.7–8.2)
Highest LDL-cholesterol level, median (IQR)	5.0 (4.2–5.6)
On statin at the time of highest total cholesterol measurement, n (%)	66 (25.4)
On high-potency statin at the time of highest total cholesterol measurement, n (%)	17 (6.5)
Ever total cholesterol >7.5 or LDL >4.9, n (%)	175 (67.3)
Number with Lp(a) record (%)	93 (35.8)
Lp(a) level, median (IQR)	156.8 (85–492)
Examined for arcus cornealis, n (%)	0
Examined for tendon xanthoma, n (%)	0

FH, familial hypercholesterolaemia; LDL, low-density lipoprotein; Lp(a), lipoprotein(a).

Table 2 compares lipid profiles and statin prescribing by the nature of genetic test result where electronic health records were available to review (n=260). Prior to the genetic diagnosis, all subgroups had raised median total and LDL-cholesterol concentrations, with those genetically confirmed to have FH having higher median values than those with other test results. The median cholesterol values in those with a VUS was similar to those with no FH-causing genetic variant and those with a high PHR score (in the 6th–10th decile). Also, a higher proportion of the genetically confirmed FH group were prescribed a statin compared with other groups. Two participants were on high-potency stains at the time of the highest ever cholesterol, both noted before the start of the study. Further comparison of absolute differences in cholesterol concentrations and statin prescribing is presented in online supplemental table 8.

**Table 2:** Current cholesterol profile and statin prescribing by reported genetic test results in 260 participants with electronic health records available

	Genetically confirmed FH (n=16)	VUS (n=9)	High PHR score (n=139*)	No genetic mutation (n=98)
Age in years, mean (SD)	55.8 (13.7)	60.2 (13.9)	56.6 (11.7)	55.9 (10.5)
Females, n (%)	10 (62.5)	9 (100)	38 (27.3)	62 (63.3)
Highest ever total cholesterol, median mmol/L (IQR)	8.6 (6.9–11.5)	7.6 (6.1–8.0)	7.7 (6.8–8.3)	7.5 (6.8–7.9)
Highest ever LDL-cholesterol, median mmol/L (IQR)	6.4 (4.4–8.5)	4.7 (3.6–5.6)	5.1 (4.4–5.7)	4.8 (4.0–5.3)
On statin at time of highest cholesterol record, n (%)	11 (68.8)	2 (22.2)	34 (24.5)	19 (19.4)
On high-potency statin at time of highest cholesterol record, n (%)	2 (12.5)	1 (11.1)	9 (6.5)	5 (5.1)
On statin at time of highest cholesterol record after study start, n (%)	6 (37.5)	2 (22.2)	19 (13.7)	10 (10.2)
On high-potency statin at time of highest cholesterol record after study start, n (%)	0	1 (11.1)	6 (4.3)	3 (3.1)
Ever total cholesterol >7.5 or LDL >4.9, n (%)	12 (75.0)	6 (66.7)	101 (72.7)	58 (59.2)
Number with Lp(a) record (%)	6 (37.5)	3	50	35
Lp(a) level, median (IQR)	157.6 (142–177)	83 (83–100)	165 (89–253.5)	150 (85–791)
Examined for arcus cornealis, n (%)	0	0	0	0
Examined for tendon xanthoma, n (%)	0	0	0	0

*This includes two patients with VUS who had high polygenic risk scores.

FH, familial hypercholesterolaemia; LDL, low-density lipoprotein; Lp(a), lipoprotein(a); PHR, polygenic hypercholesterolaemia risk; VUS, variants of unknown significance.

## Discussion

### Key findings

In patients with no previous diagnosis of FH, from a general primary care population, 26 of 283 completing genetic testing required specialist referral. Two-thirds (16) of these individuals had genetically confirmed FH, equating to 1 in 18 of those tested. A further 53% of the 283 participants tested were identified with a polygenic inherited predisposition to hypercholesterolaemia (PHR score in the 6th–10th decile).

Total cholesterol and LDL levels, as well as statin prescribing, were higher in those patients identified with newly diagnosed genetically confirmed FH. As study participants were identified using a FH case-finding tool, it is not surprising their cholesterol levels were higher than the general population. In some genetically confirmed patients, the clinical sequelae of FH, specifically arcus cornealis, was not documented in primary care records but noted on specialist clinical assessment.

### Relationship to other literature

This is the first study to introduce genetic testing in primary care to examine and confirm the genetic profile of people identified at risk of having FH from the general population, as part of routine clinical care. Previous studies on FH assessment in primary care have included intervention to identify patients at risk of FH using clinical diagnostic criteria and referring them to specialist lipid services for confirmatory diagnosis with genetic testing.^18–21^

Our previous study of cardiovascular genetic testing using postal DNA saliva collection^22^ was acceptable to patients.^23^ The current study collecting genetic blood samples in primary care appears to be similarly acceptable. As well as identifying individuals with FH-causing genetic variants, this study identified those with polygenic hypercholesterolaemia. There is increasing interest in polygenic causes of common chronic diseases, including both hypercholesterolaemia and CHD.^17 24^ However, it remains unclear how this information can be used in healthcare either as a standalone risk assessment or as part of a multifactorial risk score.^25^

In this study, we newly identified 16 patients with genetically confirmed FH from 14 general practices, based on genetic testing 283 patients from an automated primary care record search of 86 219 individuals with cholesterol readings already recorded in their medical records. This compares favourably with FH screening in children, with 20 identified with genetically confirmed FH from 92 general practices based on cholesterol testing 10 118 children at immunisation and genetic testing 92 children, over cholesterol threshold.^26^ Currently, case-finding in adults fits more closely into the infrastructure of primary care and is more resource efficient. This does not negate the value of screening children for FH, but identifying all cases of FH will require a multifaceted approach.

Following identification of index cases with genetically confirmed FH, the next step is to cascade FH screening to other relatives with around 50% of first-degree relatives affected.^9 11^ It is estimated that each index case identifies on average another 1.65 relatives with the condition.^27^ From these 14 general practices, by the end of the study period, cascade screening had already started in 8 families of genetically confirmed index cases.

### Strengths and weaknesses

Routine cholesterol measurement, as recommended by national FH guidelines, is established in primary care. In this study, we were also able to integrate genetic testing within the infrastructure of the general practice phlebotomy services using blood bottles readily available in primary care (EDTA specimen bottles most commonly used for full blood count and glycosylated haemoglobin). Furthermore, this study actively recruited diverse general practices, including 36% participating from socially deprived areas.

Given the pragmatic nature of the study and, for ethical reasons, the research team could not identify patients to recruit prior to written consent. As a consequence, practices invited some patients who did not fulfil the study eligibility criteria (FAMCAT probability threshold 1 in 500). In future studies and FH case-finding tool development, the selection of patients will be further automated to ensure only eligible patients are recruited.

### Clinical implications

The starting point for identification of FH in primary care is the availability of serum cholesterol levels. In this study, nearly 45% of the adult patients registered with the practices had a result. Periodic health checks in primary care offer the opportunity to increase the proportion of the population tested.^28^ In England, this is offered around every 5 years for the national vascular check programme.^29^ The study also demonstrated genetic testing for inherited hypercholesterolaemia can be performed in primary care. This could lead to refinement of the FH referral pathway with only those with genetically confirmed FH and those with strong clinical phenotype of FH referred to specialists. This could reduce unnecessary referrals and workload. In addition to FH, other inherited monogenic lipid disorders may be identified, for example, familial combined hyperlipidaemia. More comprehensive primary care pathways to identify several inherited lipid disorders may need to be considered.

One key challenge with current state-of-the-art genetic testing is the increased diagnostic uncertainty when analysing samples using NGS. In this study, 4% of those tested were identified with VUS. These participants also needed referral, but it could be challenging for GPs to explain the diagnostic uncertainty and need for specialist assessment to confirm FH or a ‘benign’ result. A significant proportion of these participants will then still remain classified as VUS and may return to their GPs for further clarification. Within this group, there will be patients who have the clinical features of (monogenic) FH but no definitive genetic diagnosis. In some cases, these patients will be diagnosed with polygenic hypercholesterolaemia. Specialists will still manage them aggressively with high potency statins.

As found in this study, NGS can also identify polygenic hypercholesterolaemia. Patients with these findings are unlikely to require specialist referral but this novel risk factor could contribute to multifactorial cardiovascular risk assessment, such as, Framingham or QRisk score. If this or other genetic tests are to be introduced into primary care, practitioners would need to be literate in understanding test results and explaining these findings to patients. This may require outreach specialist nurse support. A recent review recommended the need for multidisciplinary clinics involving both primary and specialist care.^30^

### Research implications

This study indicates a randomised controlled study design, measuring clinically relevant outcome measures, such as, the proportion of individuals with genetically confirmed FH achieving optimal cholesterol level, is needed to provide robust evidence on whether to introduce a combined approach using an FH case-finding tool with genetic testing in general practice. This intervention would be compared with usual practice of case-finding without genetic testing. Prior to a full trial, a pilot randomised study will be needed to estimate appropriate sample size. The cardiovascular risk associated with polygenic hypercholesterolaemia in the general population needs further exploration. The utility of incorporating the polygenic hypercholesterolaemia score into cardiovascular risk assessment tools should also be considered.

### Conclusions

Case finding for patients at increased risk of FH, combined with genetic testing, can be performed in primary care. This could help enhance the referral pathway to specialist care. This identifies genetically confirmed FH and uncertain test results and polygenic hypercholesterolaemia. The complexity of genetic test results reported indicates a need for outreach specialist support to interpret the test findings and advise on care pathways. A significant proportion of those identified with possible FH on case finding will have polygenic hypercholesterolaemia. The cardiovascular risk and management associated with this still needs to be clarified.

**Key messages**

**What is already known on this subject?**

- Familial hypercholesterolaemia (FH) is currently underdiagnosed and undertreated.
- Clinical criteria and electronic tools exist for FH case-finding in primary care.
- Genetic testing for FH is not currently offered in primary care.

**What might this study add?**

- This study demonstrates that undiagnosed FH cases can be identified from the general population in primary care, using electronic case finding and genetic testing.
- Less severe polygenic hypercholesterolaemia is also found in a large proportion of patients identified at risk of FH.

**How might this impact on clinical practice?**

- The integration of genetic testing with FH case finding in primary care electronic health records could refine current referral pathways, leading to more appropriate use of specialist services for management of genetically confirmed FH and cascade genetic testing to relatives.

## Data Availability

No data are available. We do not have consent from participants to share their data for the purposes of future research.
