# Supplementary material for: Case-finding and genetic testing for familial hypercholesterolaemia in primary care
Source: Heart. 2021 Sep 14;107(24):1956–61. doi: 10.1136/heartjnl-2021-319742 (PMC8639929; doi:10.1136/heartjnl-2021-319742)
Supplement: Supplementary data [file heartjnl-2021-319742supp001.pdf]

BMJ Publishing Group Limited (BMJ) disclaims all liability and responsibility arising from any reliance placed on this supplemental material which has been supplied by the author(s)


# Supplementary Material

## Case-finding and Genetic testing for Familial Hypercholesterolaremia in Primary Care



Qureshi N, *et al. Heart* 2021; 107:1956–1961. doi: 10.1136/heartjnl-2021-319742

**Supplementary Table 1: 12 SNP LDL-Score, showing the LDL-C-raising allele and the published raising effect (in mmol/l)**

| CHR | SNP | Gene | Minor* | Common* | Weight |
|---|---|---|---|---|---|
| 1 | rs2479409 | *PCSK9* | **G** | A | 0.052 |
| 1 | rs629301 | *CELSR2* | G | **T** | 0.15 |
| 2 | rs1367117 | *APOB* | **A** | G | 0.10 |
| 2 | rs4299376 | *ABCG8* | **G** | T | 0.071 |
| 6 | rs1564348 | *SLC22A1* | C | **T** | 0.014 |
| 6 | rs1800562 | *HFE* | A | **G** | 0.057 |
| 6 | rs3757354 | *MYLIP* | T | **C** | 0.037 |
| 11 | rs11220462 | *ST3GAL4* | **A** | G | 0.050 |
| 14 | rs8017377 | *KIAA1305* | **A** | G | 0.029 |
| 19 | rs6511720 | *LDL-R* | T | **G** | 0.18 |
| 19 | rs429358 | *APOE* | C | T | |
| 19 | rs7412 | *APOE* | T | C | |
| 19 | ε2ε2 | *APOE* | | | -0.9 |
| 19 | ε2ε3 | *APOE* | | | -0.4 |
| 19 | ε2ε4 | *APOE* | | | 0.2 |
| 19 | ε3ε3 | *APOE* | | | 0 |
| 19 | ε3ε4 | *APOE* | | | 0.1 |
| 19 | ε4ε4 | *APOE* | | | 0.2 |

* Risk alleles (LDL-C-raising) are indicated in bold. Weights are as reported in Talmud et al.[1] With SNP score of 1-3 there is a low likelihood of polygenic hypercholesterolaemia (PHC), a SNP score 6-10 there is a high likelihood of PHC and SNP score 4 and 5 indicate average likelihood.



Qureshi N, *et al. Heart* 2021; 107:1956–1961. doi: 10.1136/heartjnl-2021-319742

**Supplementary Table 2: Details of recruited practices**

| Geographical area | IMD quintile[1] | Practice list size | Ethnicity estimate[1,3] |
|---|---|---|---|
| Leicester City | 1 | 13,000 | 4.4% mixed, 6.0% Asian, 5.4% black |
| Leicester City | 1 | 13,000 | 3.6% mixed, 5.4% Asian, 7.7% black |
| Leicester City | 3 | 12,800 | 3.8% mixed, 49.0% Asian, 6.3% black, 3.2% other non-white ethnic groups |
| Northamptonshire | 4 | 48,000[2] | 1.3% mixed, 1.2% Asian, 1.3% black |
| Derbyshire | 4 | 7,305 | 1.7% non-white ethnic groups |
| West Leicestershire | 6 | 10,592 | 2.0% mixed, 15.7% Asian, 1.3% black, 1.0% other non-white ethnic groups |
| East Leicestershire | 7 | 10,695 | 1.7% mixed, 8.4% Asian, 1.5% other non-white ethnic groups |
| Derbyshire | 8 | 8,775 | 1.1% Non-white ethnic groups |
| West Leicestershire | 9 | 9,434 | 1.3% Asian, 1.3% other non-white ethnic groups |
| Derbyshire | 9 | 12,465 | 1.0% mixed, 1.1% other non-white ethnic groups |
| East Leicestershire | 10 | 12,500 | 2.4% mixed, 37.3% Asian, 1.5% black, 2.6% other non-white ethnic groups |
| West Leicestershire | 10 | 8,633 | 1.1% mixed, 2.5% Asian |
| East Leicestershire | 10 | 16,000 | 1.2% mixed, 1.7% Asian |
| Derbyshire | 10 | 10,390 | 2.0% mixed, 2.2% Asian |

*[1]Data provided National General Practice Profiles https://fingertips.phe.org.uk/profile/general-practice (1 most deprived 10 least deprived)*
*[2]This practice was a central hub that incorporated 3 general practices*
*[3]Data provided National General Practice Profiles https://fingertips.phe.org.uk/profile/general-practice*



Qureshi N, *et al. Heart* 2021; 107:1956–1961. doi: 10.1136/heartjnl-2021-319742

**Supplementary Table 3: Table of variants detected and pathogenicity designation**

| FH Result | Location | DNA Description | Protein Description | Score | Decile | (MAF) Comment |
|---|---|---|---|---|---|---|
| **LDLR** | | | | | | |
| FH Confirmed | Promoter | c.[-121T>C];[-121=] | p.[(?)];[=] | N/A | N/A | |
| FH Confirmed | Exon 1 | c.[6delG];[6=] | p.[(Trp4Glyfs*202)] | N/A | N/A | |
| FH Confirmed | Exon 4 | c.[660del];[660=] | p.[(Asp221Thrfs*44)] | N/A | N/A | |
| FH Confirmed | Exon 4 | c.[682G>T];[682=] | p.[(Glu228*)] | N/A | N/A | |
| FH Confirmed | Del e 4-14 | c.[314-?_2140+?del];[=] | p.[(?)];[=] | N/A | N/A | |
| FH Confirmed | Exon 7 | c.[986G>A];[=] | p.[(Cys329Tyr)] | N/A | N/A | |
| FH conf +VUS | Exon 7/10 | c.1399_1400delinsTA(;)948C>G | p.(Thr467Tyr);(Asn316Lys) | N/A | N/A | |
| FH Confirmed | Intron 8 | c.[1187-10G>A];[=] | p.[(?)];[=] | N/A | N/A | |
| FH Confirmed | Intron 9 | c.[1358+1G>T];[=] | p.[(?)];[=] | N/A | N/A | |
| FH confirmed | Exon 10 | c.[1444G>A];[=] | p.[(Asp482Asn)] | N/A | N/A | |
| FH Confirmed | Exon 12 | c.1816[G>T];[=] | p.[(Ala606Ser)] | N/A | N/A | |
| FH Confirmed | Del e 13+14 | N/A | N/A | N/A | N/A | |
| VUS Detected | Exon 9 | c.1263[C>A];[=] | p.[(Ser421Arg)] | 0.769 | 3 | (0.00004) Benign. Not FH causing |
| VUS Detected | Exon 16 | c.2359[G>A];[=] | p.[Val787Met] | 0.862 | 4 | (0.0005) Dam/tol. VUS |
| VUS Detected | Exon 14 | c.2072[C>T];[=] | p.[(Ser691Leu)] | 0.945 | 6 | (0.00008) Prob damaging |
| ***APOB*** | | | | | | |
| FH Confirmed | Exon 26 | c.[10580G>A];[10580=] | p.[(Arg3527Gln) | N/A | N/A | |
| FH Confirmed | Exon 26 | c.[10580G>A];[10580=] | p.[(Arg3527Gln)] | N/A | N/A | |
| FH Confirmed | Exon 26 | c.[10580G>A];[10580=] | p.[(Arg3527Gln)] | N/A | N/A | |
| VUS Detected | Exon 21 | c.3226[A>C];[=] | p.[(Ile1076Leu)];[=] | 0.796 | 3 | Novel. Prob non-Path |



Qureshi N, *et al. Heart* 2021; 107:1956–1961. doi: 10.1136/heartjnl-2021-319742

| | | | | | | |
|---|---|---|---|---|---|---|
| Hypobeta. | Exon 26 | c.10520[G>C];[=] | p.[(Arg3507Pro)] | 0.903 | 5 | (Ashkenazi 0.004). Likely benign/ not FH causing |
| VUS Detected | Exon 29 | c.[13480_13482del]; | p.[(Gln4494del)] | 1.107 | 9 | (0.0007) in vitro mild effect on LDL binding. High SNP score. Prob not FH causing |
| ***PCSK9*** | | | | | | |
| FH Confirmed | Exon 9 | c.1486[C>T];[=] | p.[(Arg496Trp)] | N/A | N/A | (0.00003) Mixed reports may be VUS |
| VUS Detected | Exon 1 | c.[118G>A];[118=] | p.[(Glu40Lys)] | 0.881 | 5 | Novel. VUS |
| VUS Detected | Exon 8 | c.[1251C>A];[1251=] | p.[(His417Gln)] | 0.874 | 4 | (African 0.003) tolerated Not FH causing |
| VUS Detected | Exon 8 | c.1207[G>A];[=] | p.[Glu403Lys] | 0.892 | 5 | (0.00003) benign |
| VUS Detected | Exon 12 | c.1979[A>C];[=] | p.[(Asp660Ala)] | 1.049 | 8 | (0.00006) prob not path. High SNP score |

**Supplementary Table 4: Summary of identified FH-causing variants and VUS found by gene**

| Gene | Number with FH causing variant | Number with VUS | Total |
|---|---|---|---|
| *APOB* | 3 | 2 | 5 |
| *LDLR* | 12 | 4 | 16 |
| *PCSK9* | 1 | 4 | 5 |
| TOTAL | 16 | 10 | 26 |

**Supplementary Table 5: Process Outcomes**

| | **Eligible patients** no. (%) |
|---|---|
| No. of individuals identified with possible FH (eligible for study) | 3,375 |
| No. of study packs despatched to practices* | 700 |
| No. of patients consenting to study participation & genetic testing (% of eligible individuals that participated in the study)^ | 336 (10.0) |
| No. who had genetic blood test (% of those invited) | 283 (85.2) |
| No. with genetically confirmed FH (% of those tested) | 16 (5.7) |
| No. with VUS (% of those tested) | 10 (3.5) |
| No. with high polygenic score (deciles 6-10: % of those tested) | 153 (54.1) |
| No. with intermediate polygenic score (deciles 4-5: % of those tested) | 55 (19.4) |
| No. with low polygenic score (deciles 1-3: % of those tested) | 59 (20.8) |
| No. of patients recommended referral to specialist. (referral rate, % of those tested) | 26 (9.2) |
| No. of patients seen by specialist in study period. (% of those referred) | 19 (73.1) |

* Fifty study participation packs (for patients) were despatched to each practice.

^ There were 3,375 individuals who were eligible to join the study but not all were invited. Rather, the 10% (336) were those eligible individuals that first consented to testing and then participated in the study



Qureshi N, *et al. Heart* 2021; 107:1956–1961. doi: 10.1136/heartjnl-2021-319742

**Supplementary Table 6: Decile profile of Polygenic Hypercholesterolemia score**

| Decile score | Number of participants |
|---|---|
| 1 | 14 |
| 2 | 21 |
| 3 | 24 |
| 4 | 32 |
| 5 | 23 |
| 6 | 36 |
| 7 | 26 |
| 8 | 36 |
| 9 | 30 |
| 10 | 25 |
| Not applicable | 16* |
| Total | 283 |

* Patients with an FH-causing variant

**Supplementary Table 7: Age-sex profile of participants with complete data for analysis (n=260) and those lost to follow-up (n=76)**

| | Lost to follow-up (n=76) | Study cohort (n=260) |
|---|---|---|
| Age, mean (SD) | 52.9 (12.4) | 56.3 (11.4) |
| Females, n (%) | 55 (72.4) | 180 (69.2) |

336 patients consented to participate in the study, 42 did not respond to genetic testing invite, 11 left practice before invite and electronic health records not available in another 23 participants.



Qureshi N, *et al. Heart* 2021; 107:1956–1961. doi: 10.1136/heartjnl-2021-319742

**Supplementary Table 8: Absolute difference in cholesterol profile and statin prescribing by reported genetic test results in 260 participants with electronic health records available**

| | **Genetically Confirmed FH** (n=16) | **High PRS** (n=139) | **No genetic mutation** [a] (n=105) | **Absolute difference** (FH mutation vs No mutation) | **Absolute difference** (High PRS vs No mutation) |
|---|---|---|---|---|---|
| Highest ever total cholesterol, mmol/l mean (SD) | 9.1 (2.6) | 7.5 (1.3) | 7.4 (1.6) | 1.70 (0.77 – 2.63) | 0.10 (-0.27 – 0.47) |
| Highest ever LDL cholesterol, mmol/l mean (SD) | 6.6 (2.1) | 4.9 (1.3) | 4.6 (1.0) | 2.00 (0.95 – 3.05) | 0.30 (-0.001 – 0.60) |
| On statin at time of highest cholesterol record, n (%) | 11 (68.8) | 34 (24.5) | 21 (20.0) | 48.8 (22.96 – 67.08) | 4.50 (-6.26 – 14.65) |
| On high-potency statin at time of highest cholesterol record, n (%) | 2 (12.5) | 9 (6.5) | 6 (5.7) | 6.80 (-4.13 – 30.52) | 0.80 (-6.10 – 6.99) |
| On statin at time of highest cholesterol record after study start, n (%) | 6 (37.5) | 19 (13.7) | 12 (11.4) | 26.1 (5.66 – 50.43) | 2.3 (-6.56 – 10.52) |
| On high-potency statin at time of highest cholesterol record after study start, n (%) | 0 | 6 (4.3) | 4 (3.8) | 3.8 (-15.70 – 9.38) | 0.5 (-5.54 – 5.81) |

[a] This includes patients with VUS as the three experts confirmed none of the VUS results were consistent with known FH genetic mutation.

**Reference**:


- Statistics with Confidence: Confidence Intervals and Statistical Guidelines. (2013). Germany: Wiley.

**Supplementary Figure 1: Histogram of SNP score profile**

*(n=267 without FH-causing variant)*

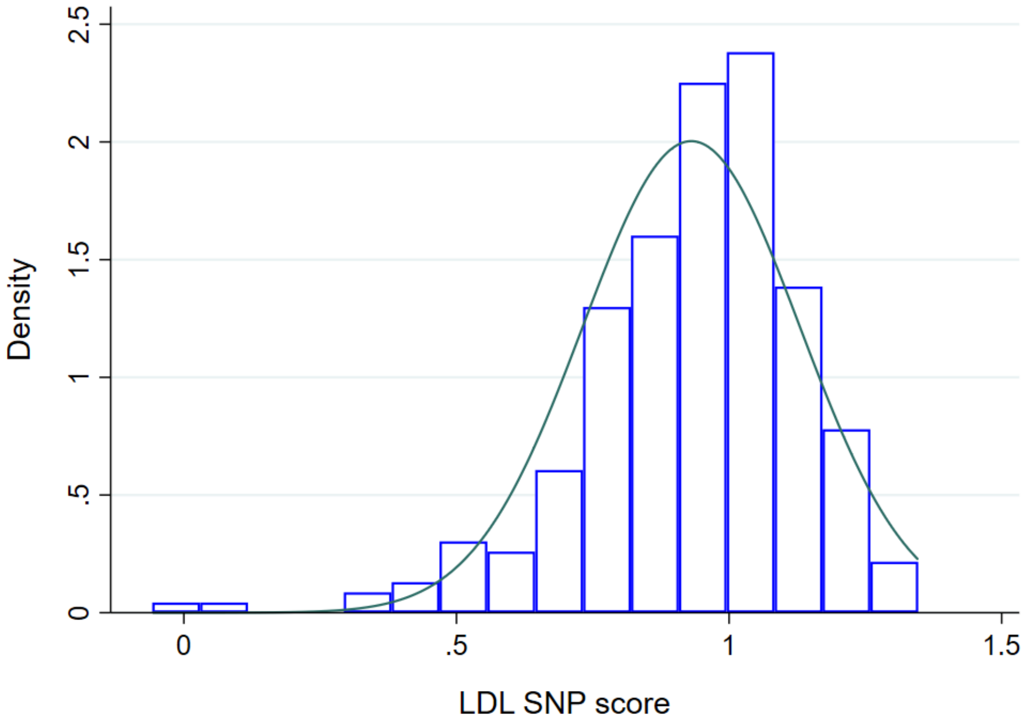


**Reference**

Talmud PJ, Shah S, Whittall R, Futema M, Howard P, Cooper JA, et al. Use of low-density lipoprotein cholesterol gene score to distinguish patients with polygenic and monogenic familial hypercholesterolaemia: a case-control study. Lancet. 2013;381(9874):1293-301.



Qureshi N, *et al. Heart* 2021; 107:1956–1961. doi: 10.1136/heartjnl-2021-319742
